# High freezing rate improves flavor fidelity effect of hand grab mutton after short-term frozen storage

**DOI:** 10.3389/fnut.2022.959824

**Published:** 2022-07-22

**Authors:** Yong-Zhao Bi, Yu-Long Luo, Rui-Ming Luo, Chen Ji, Shuang Gao, Shuang Bai, Yong-Rui Wang, Fu-Jia Dong, Xiao-Lei Hu, Jia-Jun Guo

**Affiliations:** ^1^School of Food & Wine, Ningxia University, Yinchuan, China; ^2^National R & D Center for Mutton Processing, Yinchuan, China; ^3^School of Agriculture, Ningxia University, Yinchuan, China

**Keywords:** hand grab mutton, freezing rate, eutectic point, short-term frozen storage, flavor substance, flavor fidelity

## Abstract

Taking the eutectic point as the final freezing temperature, the differences of flavor substances of in hand grab mutton (HGM) frozen at three rates of 0. 26 cm/h (−18°C), 0.56 cm/h (−40°C) and 2.00 cm/h (−80°C) were determined and analyzed. The results showed that the flavor of HGM decreased significantly after freezing. With the increase of freezing rate, the contents of aldehydes, alcohols, ketones, acids, esters, others, free amino acids and 5′-nucleotides were higher, and the content of specific substances was also generally increased. All samples from unfrozen and frozen HGM could be divided into four groups using an electronic nose based on different flavor characteristics. Seven common key aroma components were determined by relative odor activity value (ROAV), including hexanal, heptanal, octanal, nonanal, (*E*)-oct-2-enal, (2*E*,4*E*)-deca-2,4-dienal and oct-1-en-3-ol. The higher the freezing rate, the greater the ROAVs. Taste activity values calculated by all taste substances were far <1, and the direct contribution of the substances to the taste of HGM was not significant. The equivalent umami concentration of HGM frozen at −80°C was the highest. These findings indicated that higher freezing rate was more conducive to the retention of flavor substances in HGM, and the flavor fidelity effect of freezing at −80°C was particularly remarkable.

## Introduction

Tan sheep, an advantageous and characteristic breed in Ningxia of China, is famous for its meat with low “off-flavor” and good taste (1–4). In recent years, it has boarded the state banquet table many times to entertain foreign guests. With Tan sheep ribs as raw materials, hand grab mutton (HGM) is a traditional boiled meat product with national characteristics in Northwest China. At present, most of the HGM products sold in the market use the “soft can” production technology to extend the shelf life, but the high-temperature and high-pressure sterilization has seriously damaged its flavor, which is not conducive to the development of Tan sheep meat processing industry (5, 6).

The central kitchen developed earlier in America, Japan and many developed countries in Europe (7). Some traditional Western meat foods (such as steak and roast beef) have been industrialized through the central kitchen. The construction of central kitchen started late in China. Until the continuous transformation and upgrading of China's catering industry in recent years, the “industrialized” central kitchen gradually developed rapidly, organically connected the catering industry and food industry, and gradually formed a complete industrial chain (8, 9). Freezing is one of the common ways to preserve the quality of meat products (10). There is often a cold storage in the central kitchen to store semi-finished products and finished products to be delivered. Prefabricated dishes for sale can generally be transported to the terminal after short-term freezing storage. They can be eaten only after simple processing or secondary heating, effectively maintaining the original flavor of their fresh products. However, at present, the traditional commercial freezing (−18°C) has slow freezing rate and low ice crystal nucleation rate, forming a small amount of large ice crystals, which may lose more flavor substances after thawing (11–13). Therefore, flavor is still one of the difficulties to be solved urgently in the central kitchen, and the technological innovation of low-temperature flavor fidelity of semi-finished products and finished products will become an inevitable trend.

In recent years, with the development and maturity of the cold chain industry, many enterprises of China have introduced spiral quick-freezing devices (the working temperature is generally lower than −30°C) to rapidly cool the products to below −18°C in a very short time at a high freezing rate. Through rapid freezing, high nucleation rate is promoted and small ice crystals are formed in meat products, so as to minimize the loss of flavor (14–16). Eutectic point (EP) is the temperature at which all free water in the material changes from liquid to solid (17, 18). It is located behind the maximum ice crystal generation zone of the material. It is a relatively stable state reached by the product after freezing. Through high-speed freezing, the products can be quickly cooled to the EP in a very short time, and then transported to the commercial frozen storage, so as to improve the fidelity effect of product flavor and shorten the product production cycle, which can effectively reduce the cost.

At present, most scholars focus on the effect of freezing rate on the quality of raw meat and the effect of different rates frozen raw meat on the its flavor after cooking. However, there is few reports on the effect of freezing rate on the flavor substances of meat products (19–23). Therefore, the effects of three freezing rates on the differences of flavor substances in short-term frozen storage of HGM were studied in this paper. This study provides technical guidance for the frozen storage of prefabricated dishes in the central kitchen, and increases its economic value by improving the flavor fidelity effect of quick-frozen HGM. Additionally, it can be expected to provide an effective method to solve the problem of high cost caused by maintaining high freezing rate.

## Materials and methods

### Chemicals

1,2-Dichlorobenzene (99.78 g/100 g) and n-alkanes (C_6_-C_26_) of chromatographic grade were bought from Dr. Ehrenstorfer GmbH (Augsburg, Germany) and Sigma-Aldrich (St. Louis, MO, USA). Adenosine monophosphate (AMP), guanosine monophosphate (GMP), inosine monophosphate (IMP), and amino acid standards of chromatographic grade were purchased from Sigma-Aldrich (Shanghai, China). Methanol and acetonitrile were of high performance liquid chromatography (HPLC) grade and purchased from Aladdin (Shanghai, China). Perchloric acid, trichloroacetic acid, sodium hydroxide, sodium dihydrogen phosphate, and hydrochloric acid were of guaranteed reagent grade. Anhydrous citric acid, triethylamine, and acetic acid were of analytical reagent grade. All the above reagents were purchased from the Guangzhou Chemical Reagent Factory (Guangzhou, China).

### Preparation of samples

Fresh ribs were taken from approximately 6-month-old Tan sheep carcasses within 24 h after slaughter from a commercial meat company (Yanchi, Ningxia, China). The ribs were transported to the laboratory in the form of foam box and ice bag. With dirt on the surface removed, the ribs were diced into 10 cm length bars. 500 g ± 10 g ribs were weighed and soaked for 30 min each time. Then the ribs were taken out and drained, and put into the pot. And then 2 L purified water was poured (meat water ratio 1:4) and 3.5% salt (w/w, based on sheep ribs weight) were put into. There is no seasoning in the pot except salt. The ribs were boiled over high fire (2,200 W) for 30 min until boiling (1,500 m above sea level in Yinchuan, Ningxia, and the boiling point of water was 95.6°C), then skimmed the floating foam and adjusted to low fire (800 W) to continue cooking for 70 min. After cooking, the samples were put into polyethylene self-sealing bag. The samples were naturally cooled to room temperature and immediately frozen under different conditions. Four batches of HGM were prepared and stored separately at the following temperature: A) control group (Sampled and determined immediately without freezing); B) −80°C/−18°C group (After freezing at −80°C to EP, immediately transferred to −18°C and continued to store for 48 h); C) −40°C/−18°C group (After freezing at −40°C to EP, immediately transferred to −18°C and continued to store for 48 h); D) −18°C/−18°C: (After freezing at −18°C to EP, immediately transferred to −18°C and continued to store for 48 h). Before analyzing, the samples were thawed at 4°C for 24 h. After thawing, bones, the visible fat and fascia of HGM were removed. The muscle was minced at 3,000 rpm for 8 s, and analysis of volatile aroma compounds, free amino acids and taste nucleotides were performed.

### Eutectic point determination

The EP of the material was measured by differential scanning calorimetry (DSC) (DSC 800003061404, Perkin Elmer, USA). The test process was carried out by cooling. 2 ~ 8 mg mutton was placed in the aluminum crucible of DSC, protected by nitrogen, and cooled from 25 to −70°C at a cooling rate of 2°C/min. The EP of the sample was determined according to the exothermic peak value during cooling, and the results were taken as the average value of three parallel times.

### Measurement of temperature in freezing process and calculation of freezing rate

The TC-08 thermocouple data recorder (Pico, Britain) was used for multi-channel temperature measurement and recording. The thermocouple temperature probe was inserted into the geometric center of the HGM, and the temperature change was recorded every 1 min until the freezing temperature reached the EP. The temperature was measured for 6 times in parallel, and the freezing temperature curve was drawn. The freezing rate is calculated according to the method provided by the International Institute of Refrigeration (24). The calculation formula is as follows:


$$\begin{array}{l} V=\frac{\delta_{0}}{\tau_{0}} \end{array}$$

Where *V* represents freezing rate; δ_0_ represents the shortest distance between the food surface and the heat center (cm); τ_0_ represents the time required for the food surface to reach 0°C and the food center temperature to drop to 10°C lower than the food freezing point (h). In the calculation, the temperature of the central probe point of HGM was selected as the central temperature point. The determination was repeated 6 times, and δ_0_ was controlled to 1 cm.

### Electronic nose detection

Electronic nose (E-nose) analysis was performed using a PEN 3.5 E-nose (Airsense, Schwerin, Germany) according to the existing methods with minor modifications (25–27). The PEN3.5 system contains 10 metal oxide gas sensors (namely W1C, W5S, W3C, W6S, W5C, W1S, W1W, W2S, W2W, and W3S), which can detect olfactory cross-sensitive information (28–30). The response characteristics of each sensor are as follows: W1C (aromatic), W5S (broad range), W3C (aromatic), W6S (hydrogen), W5C (arom-aliph), W1S (broad-methane), W1W (sulphur-organic), W2S (broad-alcohol), W2W (sulph-chlor) and W3S (methane-aliph). 3 g of minced meat samples were put into 50 mL airtight vials and incubated in the water bath (HWS-12, Yiheng, Shanghai, China) at 25°C for 40 min. The chamber was flushed with clean air until the sensor signal returned to the baseline before testing new samples. Each sample was measured in triplicate, and the mean values were applied for further analysis.

### Analysis of volatile compounds using headspace solid phase microextraction combined with gas chromatography-mass spectrometry

Volatile compounds in the HGM were extracted using a headspace solid phase microextraction (HS-SPME) device (Supelco, Bellefone, PA, USA) with a 50/30 μm divinylbenzene/carboxen/polydimethylsiloxane (DVB/CAR/PDMS) fibre (31–33). 3 g minced meat samples were weighed into a 20 mL headspace vial. Thereafter, 4 μL of 1,2-dichlorobenzene (0.0642 μg/μL) was added as the internal standard, and then the vial was sealed tightly with a PTFE (polytetrafluoroethylene) septum. The vials were placed in water bath at 60°C for 20 min before headspace sampling to equilibrate their headspace. Then the SPME fibre, previously conditioned for 40 min at 250°C in a gas chromatograph injection port, was exposed to the headspace of a headspace vial for extraction at 60°C for 20 min. Finally, the volatile compounds were identified and quantified using a gas chromatography-mass spectroscopy (GC-MS) (Shimadzu GC-MS 2010 plus, Kyoto, Japan). The DB-WAX (30 m × 0.25 mm × 0.25 μm, Agilent, California, USA) was selected as polar analytical column. The SPME fibre was desorbed and maintained in the GC injector for 5 min at 250°C. The injection mode was splitless. The initial temperature of the column oven was isothermal for 3 min at 40°C. Thereafter, the temperature was increased to 200°C at a rate of 5°C/min and raised to 230°C at a rate of 10°C/min and held for 3 min. Helium was used as the carrier gas with a column flow rate of 2 mL/min. The MS interface temperature was 280°C, and the MS ion source temperature was 230°C. The MS was obtained using a mass selective detector in positive electron ionization mode at 70 eV. The mass scan range of m/z was set from 50 to 350 amu. The compounds were semi-quantified by comparing their mass spectra to the NIST 14 mass spectral library and by comparison of their linear retention indices (LRI), which were calculated by running C_6_-C_26_ n-alkanes under the same chromatographic conditions. Reference RI query from https://webbook.nist.gov/chemistry/. The content of each compound was calculated by comparing its area with the internal standard. Each sample was measured in triplicate, and the mean value was applied for further analysis.

### Determination of key volatile compounds

Relative odor activity value (ROAV) has been proposed to explain how to evaluate the contribution of individual volatile compounds to the overall aroma by the relative concentration of volatile compounds (34, 35). The ROAV was used to identify key volatile compounds of HGM, which range from 0 to 100. Odor thresholds in the water of volatile components were provided in the relevant literature. The calculation formula is as follows:


$$\begin{array}{l} ROAV_{i}=\frac{C_{i}}{C_{\max}}\times \frac{T_{\max}}{T_{i}}\times 100 \end{array}$$

Where *C*_i_ and *T*_i_ represent the relative content of each volatile compound and the corresponding sensory threshold (μg/L); *C*_max_ and *T*_max_ represent the relative content of the components that contribute the most to the overall flavor of the sample and the corresponding sensory threshold (μg/L).

### Measurement of free amino acids

Minced meat samples (0.5 g) were weighed in 50 mL centrifuge tubes, homogenized with 25 mL of 0.01 mol/L hydrochloric acid and followed by ultrasonic extraction for 30 min and centrifugation at 4,000 rpm for 5 min. 2 mL of the filtered liquid was accurately sucked into the centrifuge tube. After adding 2 mL of 8% sulfosalicylic acid, it was evenly mixed and allowed to stand for 15 min, and centrifuged at 10,000 r/min for 10 min. The lower aqueous phase was taken, and filtered through a membrane with 0.22 μm pore size for analysis. Amino acid analysis conditions: the chromatographic column was Hitachi special ion exchange resin (4.6 × 60 mm), and the detection wavelength was 440 nm; buffer flow rate was 35 mL/h; column temperature was 31 ~ 76°C; ninhydrin solution flow rate was 25 mL/h; injection volume was 20 μL (36, 37).

### Measurement of taste nucleotide

Minced meat samples (0.5 g) were weighed in 50 ml centrifuge tubes, added 25 mL ultrapure water, and then centrifuged at 10,000 r/min and 4°C for 20 min. The supernatant was taken in 50 mL centrifuge tubes and fixed to volume with ultrapure water. The resulting solution was filtered through a membrane with 0.22 μm pore size for analysis. HPLC conditions: the chromatographic column was TSK gel ods-80 TM (4.6 × 250 mm). The column temperature was 30°C. The ultraviolet detection wavelength was 254 nm, and the injection volume was 10 μL. The flow rate was 0.8 mL/min. Mobile phase: eluent A was 0.05 mol/L methanol, and eluent B was 0.05 mol/L potassium dihydrogen phosphate buffer with pH 5.4. After filtering with a membrane with 0.45 μm pore size, mobile phase was degassed with ultrasonic at room temperature for 30 min. The binary mobile phase was used for gradient elution separation and the detection time was 23 min. The ratio of potassium dihydrogen phosphate with time was 0 min: 0 ~ 100%; 11 min: 10% ~ 90%; 18 min: 0 ~ 100%; 23 min: 0 ~ 100% (38–40).

### Calculation of taste activity value and equivalent umami concentration

Taste activity value (TAV) value is defined as the ratio of the relative concentration of taste substance to its taste threshold, which reflects the contribution of a taste substance to the overall taste (41, 42). The formula is as follows:


$$\begin{array}{l} TAV=\frac{C}{T} \end{array}$$

Where *C* represents the absolute concentration of taste substance (mg/kg); *T* represents the threshold of taste substance (mg/kg).

The EUC is the umami taste intensity produced by the synergistic action of taste nucleotide and umami amino acid mixture, which is equivalent to the amount of single monosodium glutamate required for the umami taste intensity (43, 44). The calculation formula is as follows:


$$\begin{array}{l} Y=\sum \alpha_{i}\beta_{i}+1218\left(\sum \alpha_{i}\beta_{i}\right)\left(\sum \alpha_{j}\beta_{j}\right) \end{array}$$

Where Y represents EUC (g MSG/100 g); α_i_ represents the concentration of taste active amino acids (Asp, Glu) (g/100 g); β_i_ represents the relative freshness coefficients of umami amino acids (Asp is 0.077; Glu is 1; 1218 represents the synergy constant; α_j_ represents the concentration of taste nucleotides (5'-GMP, 5' -AMP, 5'-IMP) (g/100 g); β_j_ represents the relative freshness coefficients of taste nucleotides (5'-AMP is 0.18, 5'-IMP is 1, 5'-GMP is 2.3).

### Sensory evaluation

The sensory evaluation gets the Ningxia University's approval in advance and is performed with 12 panelists from the students and faculty of the School of Food & Wine, Ningxia University. The panelists, 6 males and 6 females aged between 18 and 40, are considered as semi-trained as they have partaken in sensory analysis of meat products for some time and are familiar with the basics of sensory evaluation. First, the HGM were stored at 50°C for temperature equilibration, then samples were cut into 1-cm^3^ pieces and mixed well before serving. Sensory analysis was undertaken in the panel booths at the university sensory laboratory (25 ± 3°C). Samples were served in plates with a glass of water and a piece of unsalted cracker to refresh the palate between samples (45). Evaluations took place in individual booths under white fluorescence light. The sensory evaluation was based on a seven-point linear scale to determine the aroma (7 = rich and strong, 1 = poor and dull), umami (7 = intense, 1 = mild), juiciness (7 = intense, 1 = mild), and overall acceptability (7 = high, 1 = low).

### Statistical analysis

Analysis of variance and mean comparison were carried out using the statistical package for the social sciences (SPSS) software (version 26.0, SPSS Inc., Chicago, IL). Differences were considered significant at *P* < 0.05. The figures were generated using Origin 2021 (OriginLab, Northampton, MA).

## Results and discussion

### Eutectic point measurement results

**Figure 1:**
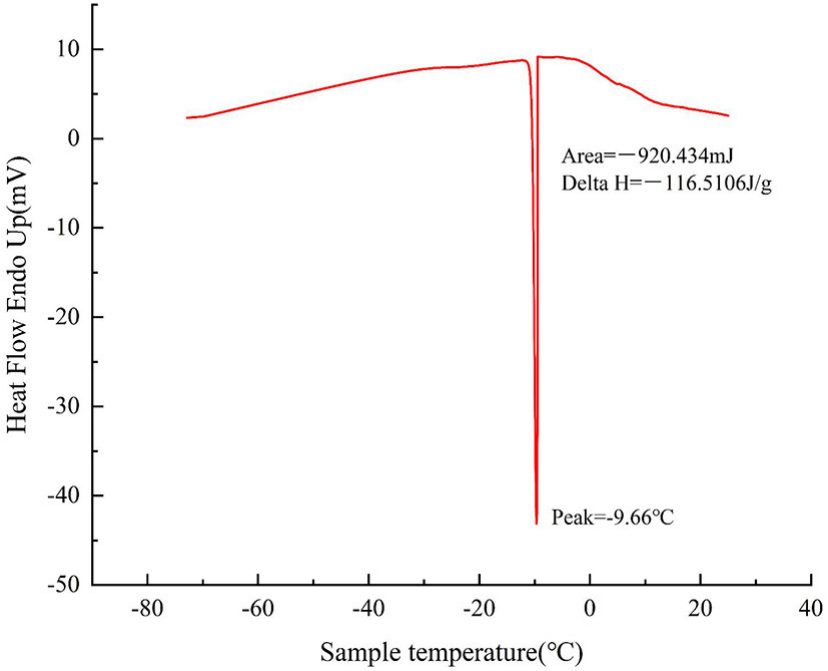
DSC cooling curve of sample.

At present, there are two methods to detect the EP of materials, namely DSC method and resistance method. DSC method uses the corresponding enthalpy change of materials when phase transition occurs, resulting in a certain exothermic or endothermic process of materials at this temperature. The EP is detected by recording the change of exothermic or endothermic process with time. During the cooling process of the sample, a large amount of free water inside the sample will crystallize and release a large amount of enthalpy heat. The DSC curve of the sample was detected as shown in Figure 1. There was only one prominent exothermic peak during the whole cooling process, so it was concluded that the EP of the sample was −9.66 ± 0.24°C.

### Effect of freezing rate on freezing curve and thawing loss rate

**Figure 2:**
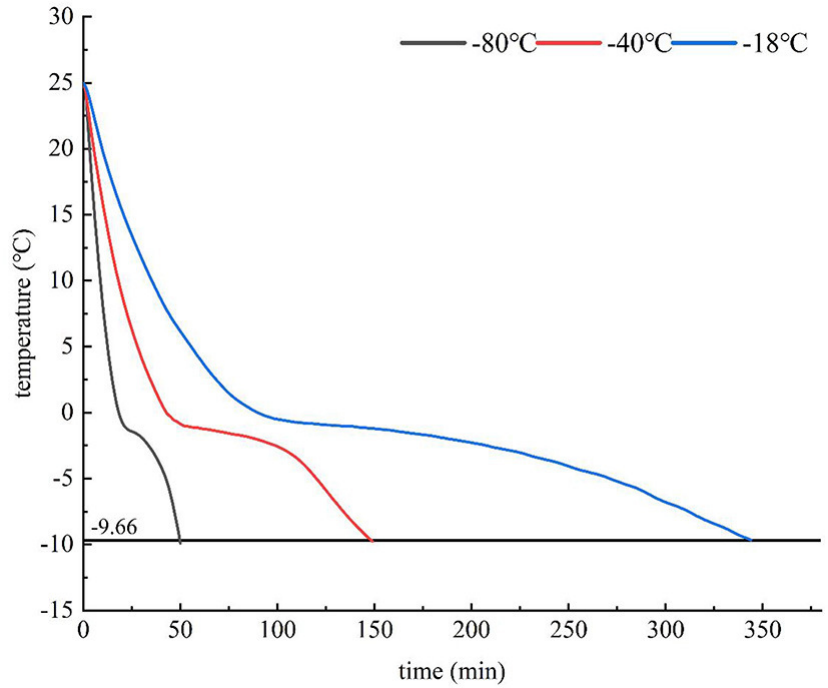
Freezing curves of different freezing temperature.

The variation law of the central temperature of HGM with time under different freezing methods was revealed in Figure 2 which is in line with the trend of general food freezing curve. The two ends drop rapidly, and the middle is relatively flat, which is the maximum ice crystal generation zone. The longer the time of passing through the maximum ice crystal formation zone, the larger the ice crystal and the more uneven the distribution, resulting in the change of cell volume, the change of colloidal properties and the mechanical damage of ice crystal, resulting in the loss of food flavor. It can be seen in the figure that the EP was located behind the maximum ice crystal generation zone. After reaching the EP, almost all the water in the mutton was coagulated into ice crystals, so that the product reached a relatively stable state after freezing. At present, in the freezing operation, we strive to pass through the maximum ice crystal generation zone as soon as possible to reduce the attenuation of product flavor. Therefore, the EP was selected as the final freezing temperature, and the samples were frozen at three rates to study the effect of freezing rate on the flavor of HGM. Jeremiah's research showed that higher freezing rate can form smaller ice crystals with large quantity and uniform distribution, which can well maintain the quality of food materials (46). It can be seen from Table 1 that the freezing rate increased gradually with the decrease of freezing temperature. According to the regulations of the International Institute of Refrigeration, the freezing rates of −18°C and −40°C were 0.26 cm/h and 0.56 cm/h respectively, which were slow freezing, and the freezing rate of −80°C was 2.00 cm/h, which was medium freezing. The time from room temperature to EP at −80°C was only 50 min, which was much shorter than −18°C and −40°C, and the thawing loss rate at −80°C was the lowest.

**Table 1:** Freezing features of different freezing temperature.

**Freezing temperature**	**Time to reach eutectic temperature/min**	**δ_0_/cm**	**τ_0_/h**	**v/cm**·**h**^−1^	**Thawing loss rate (%)**
−18°C	344	1.0	3.90	0.26	1.17 ± 0.09
−40°C	149	1.0	1.80	0.56	0.85 ± 0.05
−80°C	50	1.0	0.50	2.00	0.31 ± 0.02

### Volatile compounds profiling of hand grab mutton with different freezing rates

#### Volatile composition analysis of hand grab mutton using electronic nose

**Figure 3:**
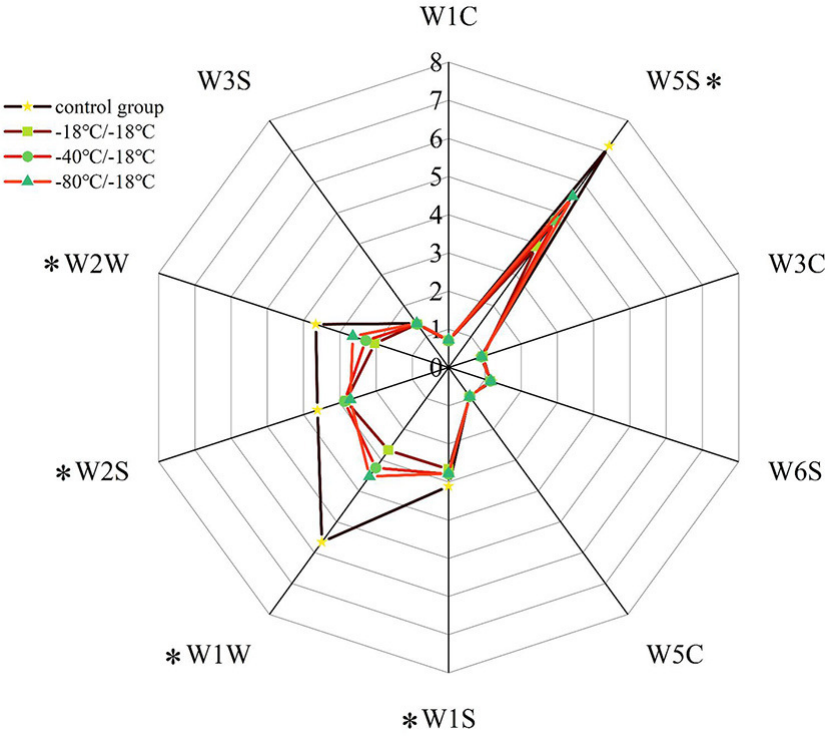
E-nose radar image of volatile flavor compounds. *Represents significant differences (*P* < 0.05).

It can be seen from Figure 3 that the odour contour curve of E-nose radar of HGM with different freezing rates was significantly different, especially between the control group and each treatment group. The signal intensity of sensors W3S, W1C, W3C, W6S, and W5C on all samples was low, and there was no significant difference between different treatment groups, indicating that the content of some aromatic compounds, ammonia, olefins and alkanes in HGM was low, and the freezing rate had little effect on it. There was no difference in W6S odour profile curve because the hydroperoxide produced by lipid oxidation has no odour. W5S, W1S, W1W, W2S, and W2W were sensitive to the response of each group of samples. Compared with the control group, the response values of the above sensors were significantly reduced in each treatment group, indicating that freezing significantly reduced the volatile flavor substances in HGM. The changes of W1S and W2S sensors showed that freezing reduced the alcohol compounds in HGM, but there was no significant difference in signal intensity between different freezing rates. The sensor W5S was sensitive to nitrogen oxides. The retention of nitrogen oxides in the −80°C/−18°C group was higher than that in the −40°C/−18°C group, and the retention of nitrogen oxides in the −18°C/−18°C group was the lowest. Sensors W1W and W2W were sensitive to sulfur compounds, and their retention law of sulfur compounds was basically consistent with the trend of W5S to nitrogen oxides. The above changes fully showed that the volatile flavor substances of HGM were seriously attenuated after freezing, but the −80°C/−18°C group could effectively reduce the loss of flavor.

**Figure 4:**
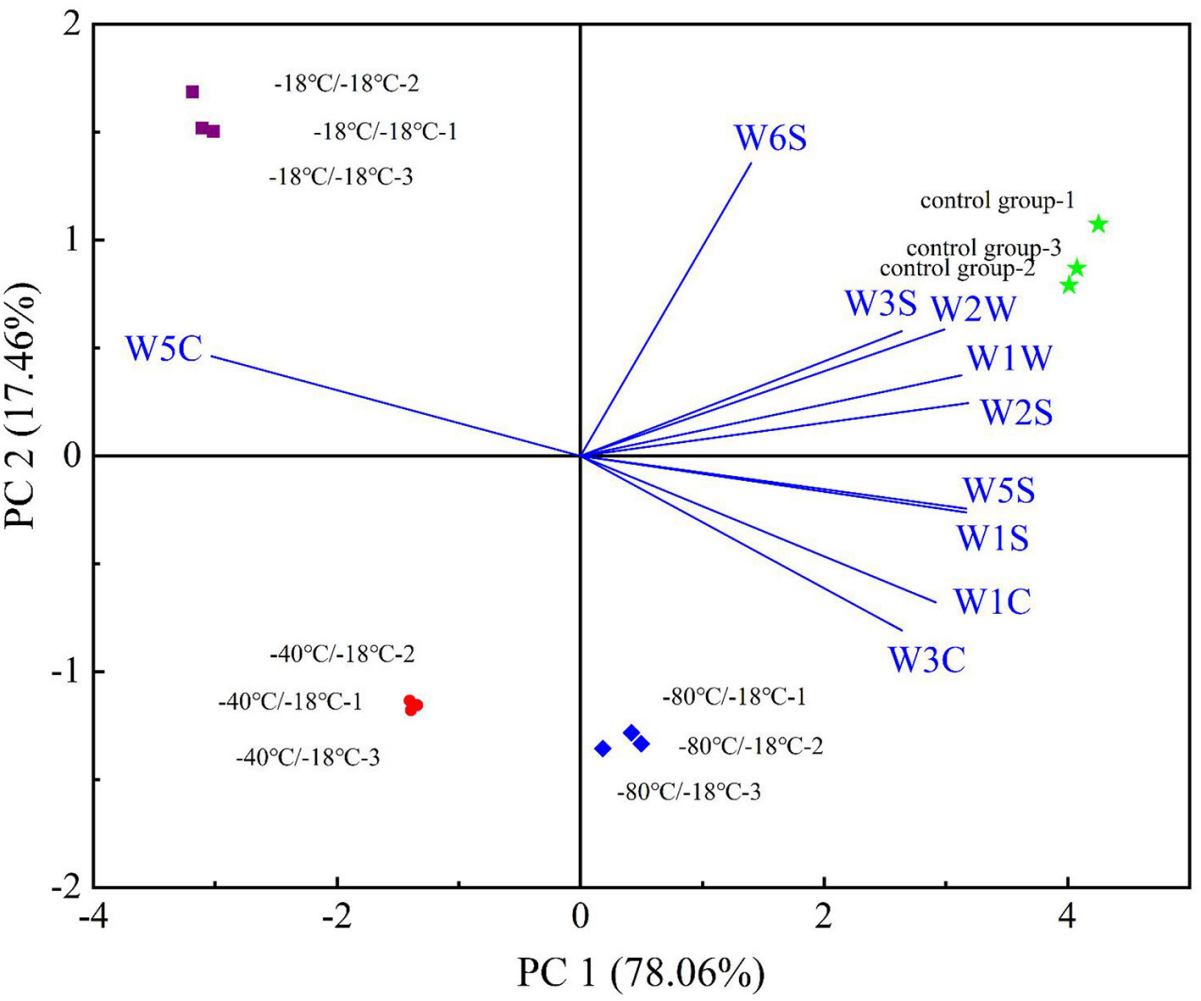
PCA biplot of E-nose.

Principal component analysis (PCA) was used to analyze the detected E-nose data. The higher the contribution rate, the better the principal component reflects the original multi-index information (47, 48). As can be seen from Figure 4, the contribution rate of PC1 was 78.06%, and that of PC2 was 17.46%. The cumulative variance contribution rate of the first two principal components was 95.52% (>95%), indicating that most of the odour information of the sample was covered by the first two principal components (26). According to Figure 4, each group of samples can be easily divided into four groups. When the samples overlap or are close, it indicates that they produce similar volatile flavor substances. In the biplot, W2S, W3S, W6S, W1W, and W2W were related to the samples of the control group, W5C was related to the −18°C/−18°C group, W1C, W3C, W1S, and W5S were related to the −80°C/−18°C group, and the −40°C/−18°C group was negative on PC1 and PC2, and there was no sensor associated with it. The analysis combined with E-nose radar fingerprint showed that the nitrogen oxides, aromatic compounds and alcohol sulfur components in HGM at different freezing rates changed significantly, while alkanes, hydrides and ammonia compounds were not affected by freezing rates. Therefore, E-nose could effectively distinguish each group of HGM, but it could not characterize the change law of specific substances after freezing.

#### Volatile components analysis of hand grab mutton by headspace solid phase microextraction combined with gas chromatography-mass spectrometry

**Figure 5:**
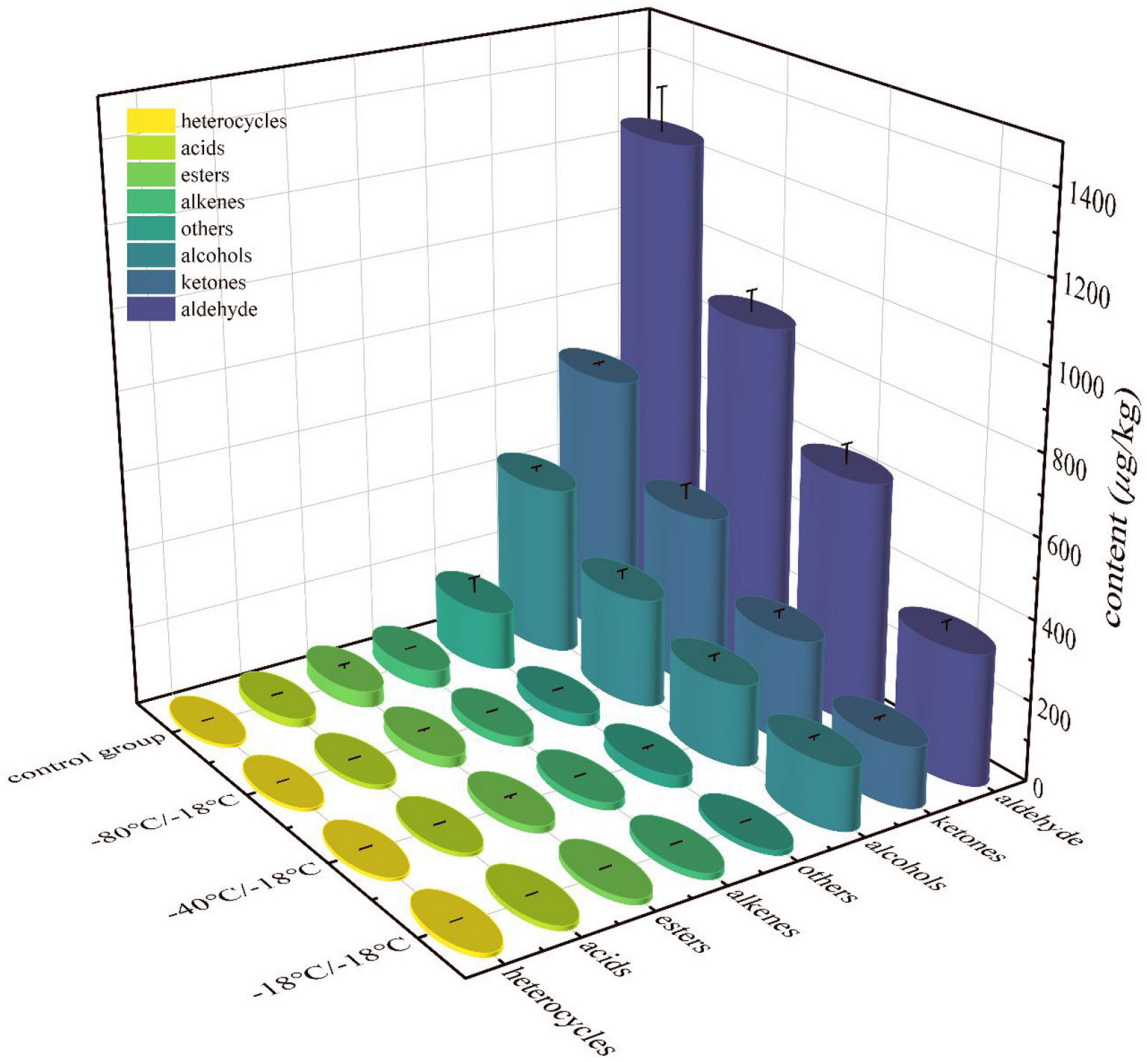
Total contents of various volatile flavor substances of HGM with different freezing rates.

The volatile flavor compounds formed in the cooking process of HGM were very complex, which were mainly produced by lipid oxidation, Maillard reaction and lipid-Maillard interaction. It can be seen from Figure 5 that aldehydes, alcohols and ketones always dominated before and after frozen storage, and were the main volatile substances in HGM. The total contents of aldehydes, alcohols, ketones, acids, esters and other substances in the three treatment groups were lower than those in the control group, indicating that short-term frozen storage led to a large loss of volatile aroma components in mutton. The changes of E-nose odour contour curve (Figure 3) were corresponded to this result.

It can be seen from Table 2 that 44 kinds (control group), 43 kinds (−18°C/−18°C), 47 kinds (−40°C/−18°C) and 48 kinds (−80°C/−18°C) were detected in the four groups of samples. The contents of aldehydes, alcohols and ketones accounted for about 90% of the total volatile components of each group of samples, which were the main volatile components of HGM. Among the aldehydes, hexanal, heptanal, octanal, nonanal, benzaldehyde, (*Z*)-2-heptanal, (*E*)-oct-2-enal, (2*E*,4*E*)-deca-2,4-dienal and tetradecanal were the main aldehydes. The oxidation and degradation of fat and the Strecker degradation of amino acids were the main sources of these substances. The threshold of aldehydes was generally low, which made an important contribution to the overall characteristics of odour (49). Aldehydes in −18°C/−18°C, −40°C/−18°C and −80°C/−18°C groups decreased by 73.53, 50.75, and 29.14% respectively. The alcohols detected in the sample mainly came from fat oxidation, and most of them were saturated alcohols (50). The olfactory threshold of saturated alcohols was high, so they contribute less to the overall flavor of HGM (51). 1-octene-3-ol was the highest content of detected alcohols. It belonged to unsaturated alcohols and had a low threshold. It could be formed by eicosapentaenoic acid catalyzed by 15-lipoxygenase and arachidonic acid catalyzed by 12-lipoxygenase (52). It was an important component of mutton aroma (53, 54). Alcohol in −18°C/−18°C, −40°C/−18°C and −80°C/−18°C group decreased by 62.81, 52.28, and 35.78% respectively. Ketones were not only the product of Maillard reaction, but also the result of fat oxidation. The reaction of lysine, arginine, leucine, methionine with glucose or fructose was the main way to produce ketones (55). The highest content of ketones detected in the control group was octane-2,3-dione, followed by 3-hydroxy-2-butanone. 3-Hydroxy-2-butanone and octane-2,3-dione were common methyl ketones in the mutton. They were related to the degree of lipid oxidation. They could be produced by the decomposition of amino acids or by β-Oxidation of fatty acids produced after heat treatment of triglycerides (56, 57). Other ketone substances such as octan-3-one, pentane-2,3-dione, undecan-2-one, dihydro-5-methyl-2(3H)-furanone, 6-pentyloxan-2-one, (*Z*)-6,10-dimethyl-5,9-undediene-2-one, (3*E*,5*E*)-nona-3,5-dien-2-one, etc. had low contents or were only detected in some samples of the treatment group. The reason might be the differences of raw meat quality or the polymerization of fat cleavage products in frozen storage. The aroma threshold of ketones was high, and generally had little contribution to the overall flavor of mutton. Compared with the control group, ketones in −18°C/−18°C group, −40°C/−18°C group and −80°C/−18°C group decreased by 77.02, 60.59, and 34.56% respectively.

**Table 2:** Volatile flavor compounds of HGM with different freezing rates.

**Category**	**No.**	**Compounds**	**Threshold (****43****) (**μ**g/kg)**	**LRI**	**Reference**	**Concentration (**μ**g/kg)**			
						**Control group**	**−18**°**C/**−**18**°**C**	**−40**°**C/**−**18**°**C**	**−80**°**C/**−**18**°**C**
Aldehydes (20)	1	Hexanal	7.5	1,069	1,078	648.10 ± 85.25^d^	225.17 ± 13.82^a^	459.34 ± 28.70^b^	565.70 ± 42.11^c^
	2	Heptanal	10	1,187	1,181	151.19 ± 3.98^d^	22.13 ± 2.74^a^	33.67 ± 5.66^b^	73.64 ± 0.49^c^
	3	Octanal	0.1	1,280	1,287	125.94 ± 3.56^d^	21.54 ± 4.70^a^	36.49 ± 3.76^b^	55.92 ± 0.77^c^
	4	Nonanal	3.5	1,372	1,390	250.7 ± 17.67^d^	40.70 ± 0.83^a^	52.05 ± 9.63^b^	135.58 ± 7.98^c^
	5	Decanal	5	1,485	1,498	—	0.74 ± 0.02^a^	0.97 ± 0.25^b^	3.32 ± 0.04^c^
	6	Benzaldehyde	300	1,520	1,508	29.67 ± 0.11^c^	8.15 ± 0.72^a^	17.62 ± 1.49^b^	17.45 ± 1.22^b^
	7	Benzene-1,3-dicarbaldehyde	UN	1,347	2,341	—	—	0.19 ± 0.07^a^	0.61 ± 0.27^b^
	8	(*Z*)-Hept-2-enal	10	1,311	1,319	12.64 ± 0.22^c^	2.09 ± 0.10^a^	2.08 ± 0.05^a^	6.62 ± 0.10^b^
	9	(*E*)-Hept-2-enal	17	1,533	1,542	—	—	—	0.38 ± 0.09^a^
	10	(*E*)-Non-2-enal	0.07	1,159	1,162	—	—	0.95 ± 0.31^a^	—
	11	(*E*)-Oct-2-enal	0.2	1,441	1,434	16.22 ± 0.47^c^	3.32 ± 0.19^a^	3.41 ± 0.13^a^	10.16 ± 0.76^b^
	12	(*Z*)-Hept-4-enal	0.04	1,235	1,237	—	0.15 ± 0.01^a^	—	—
	13	(*E*)-Non-4-enal	40	1,453	1,458	—	0.79 ± 0.04^a^	0.75 ± 0.37^a^	2.65 ± 0.23^b^
	14	(2*E*,4*E*)-Deca-2,4-dienal	0.05	1,827	1,805	3.08 ± 0.72^c^	1.49 ± 0.20^a^	2.29 ± 0.15^b^	3.15 ± 0.46^c^
	15	(2*E*,4*E*)-Nona-2,4-dienal	0.06	1,715	1,702	—	0.95 ± 0.10^a^	—	—
	16	(2*E*,6*Z*)-Nona-2,4-dienal	0.02	1,587	1,591	—	—	—	0.23 ± 0.00^a^
	17	Undec-2-enal	0.14	1,747	1,755	—	—	—	0.67 ± 0.09^a^
	18	Dodecanal	1.07	1,705	1,709	0.63 ± 0.38^a^	—	—	—
	19	Tridecanal	10,000	1,822	1,824	—	—	0.28 ± 0.05^a^	0.73 ± 0.39^a^
	20	Tetradecanal	UN	1,919	1,924	3.33 ± 0.47^c^	1.44 ± 0.92^bd^	1.39 ± 0.37^d^	2.92 ± 0.64^abc^
Alcohols (22)	1	Heptan-1-ol	520	1,473	1,461	4.39 ± 0.72^a^	—	—	—
	3	Butan-1-ol	10,000	1,138	1,150	1.35 ± 0.06^b^	0.57 ± 0.04^a^	—	—
	4	Pentan-1-ol	5,000	1,249	1,255	43.17 ± 0.12^d^	17.32 ± 0.59^a^	22.12 ± 2.16^b^	33.84 ± 1.34^c^
	5	Hexan-1-ol	200	1,366	1,359	42.27 ± 5.28^d^	6.36 ± 0.13^a^	10.91 ± 1.45^b^	17.11 ± 0.12^c^
	6	Octan-1-ol	54	1,565	1,554	13.18 ± 0.28^d^	3.37 ± 0.30^a^	5.55 ± 0.48^b^	10.86 ± 1.34^c^
	7	Nonan-1-ol	2	1,664	1,666	—	0.37 ± 0.06^a^	—	—
	8	Phenylmethanol	5,500	1,870	1,877	1.07 ± 0.01^d^	0.54 ± 0.02^a^	0.78 ± 0.02^b^	0.96 ± 0.07^c^
	9	Butane-1,4-diol	2,700,000	1,868	1,861	0.76 ± 0.03^c^	0.21 ± 0.04^a^	0.19 ± 0.06^a^	0.36 ± 0.01^b^
	10	Oct-1-en-3-ol	2.00	1,427	1,456	295.00 ± 2.79^d^	117.30 ± 5.84^a^	153.62 ± 11.36^b^	195.15 ± 17.86^c^
	11	Pent-1-en-3-ol	3,000	1,144	1,157	—	4.37 ± 0.75^a^	—	—
	12	Non-1-en-3-ol	UN	1,539	1,555	—	1.36 ± 0.22^a^	1.78 ± 0.32^ab^	2.76 ± 0.91^b^
	13	(2*R*,3*R*)-Butane-2,3-diol	1,000,000	1,553	1,544	2.18 ± 0.06^a^	—	—	—
	14	2-Ethylhexan-1-ol	UN	1,502	1,484	6.19 ± 2.97^b^	3.50 ± 0.72^a^	—	—
	15	3-Methylheptan-2-ol	UN	1,655	1,676	0.30 ± 0.02^a^	—	—	—
	16	(*Z*)-Oct-2-en-1-ol	50	1,550	1,547	15.68 ± 0.20^c^	4.01 ± 1.18^a^	8.74 ± 0.88^b^	10.79 ± 1.26^b^
	17	Dodecan-1-ol	3	1,961	1,980	—	—	—	0.25 ± 0.15^a^
	18	4-Methoxy-4-methylpentan-2-ol	UN	1,780	1,787	1.02 ± 0.57^a^	—	—	—
	19	(*Z*)-3,7-Dimethylocta-2,6-dienyl	UN	1,811	1,806	0.75 ± 0.32^a^	—	—	—
	20	Hexadecan-1-ol	UN	2,370	2,379	0.54 ± 0.24^a^	—	—	—
	21	2,4-dimethyl-Cyclohexanol	UN	1,017	1,032	—	—	—	2.91 ± 0.15^a^
	22	2-(dodecyloxy)-Ethanol	UN	1,138	1,135	0.38 ± 0.10^a^	—	—	—
Ketones (10)	1	Octan-3-one	1,000	1,249	1,261	—	0.57 ± 0.02^a^	—	—
	2	Octane-2,3-dione	UN	1,321	1,325	664.12 ± 7.50^d^	152.59 ± 9.42^a^	257.70 ± 20.53^b^	425.17 ± 38.29^c^
	3	Pentane-2,3-dione	5	1,055	1,062	—	—	4.25 ± 0.45^a^	10.06 ± 0.85^b^
	4	3-Hydroxy-2-butanone	10,000	1,291	1,280	5.00 ± 0.11^a^	—	—	—
	5	Tridecan-2-one	500	1,804	1,816	0.80 ± 0.28^b^	0.20 ± 0.03^a^	0.66 ± 0.14^b^	—
	6	Undecan-2-one	30	1,612	1,599	—	0.55 ± 0.17^a^	0.63 ± 0.05^a^	1.39 ± 0.23^b^
	7	dihydro-5-methyl-2(3H)-Furanone	UN	1,621	1,619	—	—	—	1.97 ± 0.19^a^
	8	6-Pentyloxan-2-one	UN	2,199	2,190	—	—	0.05 ± 0.00^a^	—
	9	(*Z*)-6,10-dimethylundeca-5,9-dien-2-one	UN	1,810	1,813	0.28 ± 0.06^b^	0.12 ± 0.00^a^	0.22 ± 0.00^b^	—
	10	(3*E*,5*E*)-nona-3,5-dien-2-one	UN	1,865	1,879	—	—	0.60 ± 0.28^a^	—
Acids (9)	1	Acetic acid	5,500	1,473	1,452	8.66 ± 0.50^b^	2.77 ± 0.29^a^	2.39 ± 0.80^a^	3.74 ± 0.82^a^
	2	Pentanoic acid	5,000	1,765	1,744	—	—	—	1.11 ± 0.06^a^
	3	Hexanoic acid	1,500	1,833	1,849	—	6.91 ± 0.38^a^	5.80 ± 0.87^a^	—
	4	Heptanoic acid	3,000	1,947	1,960	1.54 ± 0.06^c^	0.76 ± 0.12^a^	0.55 ± 0.09^a^	0.94 ± 0.05^b^
	5	Octanoic acid	800	2,065	2,070	1.25 ± 0.04^c^	0.99 ± 0.10^b^	0.66 ± 0.04^a^	1.09 ± 0.05^b^
	6	Nonanoic acid	1,500	2,180	2,169	3.81 ± 0.01^a^	—	—	3.39 ± 0.54^a^
	7	Decanoic acid	120,000	2,265	2,279	—	0.20 ± 0.00^a^	—	—
	8	Dodecanoic acid	UN	2,486	2,502	0.36 ± 0.05^a^	—	—	—
	9	Pentadecanoic acid	UN	2,801	2,819	4.99 ± 1.71^a^	—	—	—
Alkenes (3)	1	3,3-Dimethylhepta-1,6-diene	UN	1,459	1,450	—	0.34 ± 0.03	—	—
	2	8-Methylundec-1-ene	UN	1,198	1,124	—	—	0.19 ± 0.03^a^	0.38 ± 0.04^b^
	3	Styrene	120	1,260	1,254	44.86 ± 0.34^c^	15.21 ± 1.97^a^	18.01 ± 1.32^a^	23.53 ± 3.51^b^
Esters (5)	1	Oxolan-2-one	UN	1,604	1,595	—	0.27 ± 0.01^a^	—	—
	2	Butyl prop-2-enoate	UN	1,173	1,189	5.24 ± 0.37^a^	—	—	—
	3	Diethyl benzene-1,2-dicarboxylate	UN	2,397	2,401	20.75 ± 10.16^b^	4.83 ± 0.51^a^	6.10 ± 1.25^a^	11.18 ± 2.84^b^
	4	n-Caproic acid vinyl ester	UN	1,258	1,244	14.12 ± 1.19^c^	8.82 ± 2.04^a^	9.81 ± 6.17^abc^	14.86 ± 1.41^b^
	5	2-oxo-Nonanoic acid, methyl ester	UN	2,274	2,290	—	—	0.59 ± 0.19^a^	1.39 ± 0.18^b^
Heterocycles (1)	1	2-pentyl-Furan	4.8	1,237	1,235	6.75 ± 0.32^a^	10.79 ± 0.20^c^	7.65 ± 1.79^ab^	7.73 ± 0.49^b^
Others (12)	1	Phenol	5,500	2,021	2,008	—	0.55 ± 0.02^a^	—	—
	2	Hexanamide	280	1,113	1,135	—	—	0.24 ± 0.06^a^	0.36 ± 0.01^b^
	3	Toluene	500	1,059	1,043	138.10 ± 38.76^b^	—	23.23 ± 4.75^a^	—
	4	Ethylbenzene	2205.25	1,139	1,126	—	12.05 ± 0.79^a^	—	19.74 ± 0.33^b^
	5	1,4-Xylene	UN	1,156	1,164	—	—	3.01 ± 0.32^a^	—
	6	1-Methyl-4-propan-2-ylbenzene	UN	1,259	1,272	—	—	—	1.43 ± 0.26^a^
	7	2,6-Ditert-butyl-4-methylphenol	UN	1,880	1,902	—	—	0.58 ± 0.17^a^	0.65 ± 0.11^a^
	8	Nonadecane	UN	275	282	—	—	0.13 ± 0.01^a^	—
	9	Undecane	1,170	167	180	7.44 ± 0.86^c^	—	2.15 ± 0.72^a^	4.08 ± 0.72^b^
	10	Dodecane	2,040	205	200	—	—	—	2.36 ± 0.25^a^
	11	Tridecane	2,140	227	219	—	—	1.31 ± 0.28^a^	—
	12	Tetradecane	1,000	240	236	2.49 ± 0.95^b^	—	0.45 ± 0.08^a^	1.95 ± 0.59^b^

*All data are expressed as the means ± standard error of triple measurements*.

a−d *Means within the same line with different uppercase letters differ significantly (P < 0.05)*.

*“—” represents volatile compounds not detected*.

*UN, relevant threshold not found*.

Acids were produced when fat was oxidized or hydrolyzed into low-grade fatty acids. Due to their low content and relatively high threshold, their contribution to the overall flavor of the sample was small (58). In the control group, only three esters were detected, including butyl prop-2-enoate, diethyl benzene-1,2-dicarboxylate and n-caproic acid vinyl ester. Protein hydrolysis, glycolysis and fat oxidation were important pathways for the formation of esters (59). 2-pentyl-furan was the only heterocyclic compound detected, which was oxygen heterocyclic compound. As an indicator of lipid oxidation in meat products, it was detected in all samples. Most furan compounds had strong meat flavor, mainly produced by thiamine degradation, caramelization and carbohydrates degradation, while 2-pentyl-furan presented fruit aroma (60, 61). Among other compounds, alkanes mainly came from lipid oxidation. The contents of undecane and tetradecane were high, but their threshold was also high. Therefore, the overall flavor contribution for HGM was small. As a harmful substance, toluene mainly presented aromatic flavor.

Among the volatile flavor substances shown in Table 2, the contents of hexanal, heptanal, octanal, nonanal, benzaldehyde, (2*E*,4*E*)-deca-2,4-dienal, pentan-1-ol, hexan-1-ol, phenylmethanol, 1-octene-3-ol, (*Z*)-oct-2-en-1-ol, 2,3-octadione and undecane showed the rule of control group > −80°C/−18°C group > −40°C/−18°C group > −18°C/−18°C group (*P* < 0.05). With the increase of freezing rate, the retention of (*Z*)-2-heptanal, (*E*)-oct-2-enal, tetradecanal, butan-1-ol, octan-1-ol, nonanoic acid, styrene, diethyl benzene-1,2-dicarboxylate and toluene increased significantly (*P* < 0.05). The contents of dodecanal, heptan-1-ol, 2-ethyl- hexan-1-ol and tetradecane decreased significantly after freezing (*P* < 0.05). In addition, (2*R*,3*R*)-butane-2,3-diol, 3-methylheptan-2-ol, 4-methoxy-4-methylpentan-2-ol, (*Z*)-3,7-dimethyl-2,6-octadiene-1-ol, hexadecan-1-ol, 2-(dodecyloxy)-ethanol, 3-hydroxy-2-butanone, dodecanoic acid, pentadecanoic acid and butyl prop-2-enoate were only present in the control group and were not detected after freezing-thawing.

Part of the reason for the above results was that the water in the mutton condensed into ice crystals during freezing, and the water lost after thawing, which took away some volatile flavor substances (62, 63). Most aroma substances had high activity in aqueous liquid phase system, so they had high relative volatility. The relative volatility of volatile flavor substances relative to water determined the relative proportion of volatile components and water (64). The size of ice crystals formed by freezing water in mutton was different with different freezing rates (65). The higher the freezing rate, the smaller, more uniform and denser ice crystals would be formed in the mutton. The larger the ice crystals formed by freezing, the greater the damage to the microstructure of the mutton, resulting in serious water loss, which was also an important reason for the different degree of water loss after thawing. The change law of the thawing loss rate mentioned above was consistent with it. The increase of freezing water loss led to the loss of more volatile flavor substances, resulting in the decline of retention. Therefore, the retention and escape of water in HGM were closely related to the embedding and release of volatile flavor substances. When HGM frozen to the EP at a high rate, it could effectively curb the loss of volatile flavor substances.

In addition, the changes in the structure and properties of macromolecules (protein and fat) in mutton might also affect the retention of volatile flavor substances (65, 66). Different protein components had been proved to have different binding affinity for flavor substances. Proteins with higher contents of lysine, arginine and cysteine may show higher flavor binding ability because they involve more covalent bonds (67). Due to the differences of physicochemical properties of flavor substances and the changes of protein structure and properties caused by freezing process, such as the exposure of protein hydrophobic groups, the adsorption and binding ability of proteins to volatile flavor substances were different after freezing (67). Additionally, freezing may also destroy the interaction between protein molecules and water and lipid molecules. In the process of meat cooking, the physical crosslinking of macromolecules formed a high-density network, which inhibited the movement of water molecules and macromolecular substances and kept the whole system stable. The formation of ice crystals in the freezing process broken the stable network structure, resulting in the loss of water diversion and the change of physical and chemical properties of macromolecular substances, which aggravated the attenuation of volatile flavor substances.

#### Analysis of key volatile flavor compounds

The volatile flavor compounds of HGM with different freezing rates were analyzed by ROAV method. The greater the ROAV, the greater its contribution to the mutton aroma. The components with ROAV > 1 are key flavor compounds, the components with 0.1 < ROAV < 1 are modified flavor compounds, and the components with ROAV < 0.1 are potential flavor compounds (68, 69).

It can be seen from Table 3 that there were nine key odour compounds with ROAV > 1 in the volatile flavor substances produced by each group of samples, namely: hexanal, heptanal, octanal, nonanal, (*E*)-non-2-enal, (*E*)-oct-2-enal, (2*E*,4*E*)-deca-2,4-dienal, (2*E*,4*E*)-nona-2,4-dienal and 1-octene-3-ol, which is similar with other studies on mutton products (64–66). These key odour compounds included eight aldehydes and one alcohol, indicating that aldehydes played a leading role in the overall flavor of HGM. Among them, hexanal, heptanal, octanal, nonanal, (*E*)-oct-2-enal, (*E, E*)-2,4-decendienal and 1-octene-3-ol were detected in the four groups of samples, and their ROAVs were the highest in the control group, and all decreased significantly after frozen storage (*P* < 0.05), indicating that the aroma loss of HGM was serious after short-term frozen storage. The ROAVs of these substances were the highest in the −80°C/−18°C group (*P* < 0.05). To sum up, the retention effects of key volatile flavor compounds in HGM at three freezing rates were −80°C/−18°C group > −40°C/−18°C group > −18°C/−18°C group.

**Table 3:** Information on characteristic volatile compounds of HGM with different freezing rates.

**Serial number**	**Compounds**	**Threshold (**μ**g/kg)**	**Control group**		**−18**°**C/**−**18**°**C**		**−40**°**C/**−**18**°**C**		**−80**°**C/**−**18**°**C**	
			**Concentration** (μ**g/kg)**	**ROAV**	**Concentration** **(**μ**g/kg)**	**ROAV**	**Concentration** **(**μ**g/kg)**	**ROAV**	**Concentration** **(**μ**g/kg)**	**ROAV**
1	Hexanal	7.50	648.10	6.86^d^	225.17	2.38^a^	459.34	4.86^b^	565.70	5.99^c^
2	Heptanal	10.00	151.19	1.20^d^	22.13	0.18^a^	33.67	0.27^b^	73.64	0.58^c^
3	Octanal	0.10	125.94	100^d^	21.54	17.10^a^	36.49	28.97^b^	55.92	44.40^c^
4	Nonanal	3.50	250.70	5.69^d^	40.70	0.92^a^	52.05	1.18^b^	135.58	3.08^c^
5	(*E*)-Non-2-enal	0.07	—	0.00^a^	—	0.00^a^	0.95	1.08^b^	—	0.00^a^
6	(*E*)-Oct-2-enal	0.20	16.22	6.44^c^	3.32	1.32^a^	3.41	1.35^a^	10.16	4.03^b^
7	(2*E*,4*E*)-Deca-2,4-dienal	0.05	3.08	4.89^c^	1.49	2.37^a^	2.29	3.64^b^	3.15	5.00^c^
8	(2*E*,4*E*)-Nona-2,4-dienal	0.06	—	0.00^a^	0.95	1.26^b^	—	0.00^a^	—	0.00^a^
9	Oct-1-en-3-ol	2.00	295.00	11.71^d^	117.30	4.66^a^	153.62	6.10^b^	195.15	7.75^c^

a−d *Means within the same line with different uppercase letters differ significantly (P < 0.05)*.

### Non-volatile compounds of hand grab mutton with different freezing rates

#### Free amino acids analysis of hand grab mutton

Free Amino Acid (FAA) is the final product of protein degradation. It not only promotes the production of taste, but also participates in Maillard reaction to form volatile substances such as aldehydes, ketones, alcohols and esters. These substances can also react with lipid oxidation products to form unique flavor (70). The composition of FAAs is not only related to the nutritional characteristics of HGM, but also closely related to its flavor characteristics. It can be seen from Table 4 that there were 16 kinds of FAA (Asp, Thr, Ser, Glu, Gly, Ala, Cys, Val, Met, Ile, Leu, Tyr, Phe, Lys, His, Arg) and 7 kinds of essential amino acids (Val, Leu, Ile, Phe, Met, Lys, Thr) in the HGM with different freezing rates. ASP and Glu were the main umami amino acids in HGM, while Gly, Ala, Ser, and Pro were the main sweet amino acids.

**Table 4:** Free amino acids of HGM with different freezing rates.

**Taste attributes**	**Compounds**	**Concentrations in HGM (mg/kg)**			
		**Control group**	**−18**°**C/**−**18**°**C**	**−40**°**C/**−**18**°**C**	**−80**°**C/**−**18**°**C**
Umami	Asp	2.47 ± 0.05^a^	1.62 ± 0.19^b^	1.70 ± 0.24^b^	2.35 ± 0.12^a^
	Glu	12.86 ± 0.55^ab^	12.16 ± 0.30^ab^	11.82 ± 0.97^b^	14.13 ± 1.81^a^
TUAA		15.33 ± 0.49^ab^	13.78 ± 0.4^b^	13.52 ± 0.99^b^	16.48 ± 1.58^a^
Sweetness	Ser	1.08 ± 0.14^a^	0.85 ± 0.18^a^	0.91 ± 0.09^a^	1.10 ± 0.12^a^
	Ala	2.82 ± 0.10^a^	2.71 ± 0.28^a^	2.93 ± 0.34^a^	2.65 ± 0.06^a^
	Gly	9.37 ± 0.05^a^	7.92 ± 0.14^c^	8.75 ± 0.09^b^	9.27 ± 0.14^a^
	Thr^*^	3.16 ± 0.30^a^	1.59 ± 0.17^b^	1.57 ± 0.05^b^	1.82 ± 0.08^b^
TSAA		16.43 ± 0.48^a^	13.07 ± 0.63^c^	14.16 ± 0.47^bc^	14.84 ± 0.33^b^
Bitterness	Cys	7.80 ± 0.11^a^	5.79 ± 0.09^b^	7.82 ± 0.15^a^	7.87 ± 0.10^a^
	Met^*^	6.83 ± 0.10^a^	6.19 ± 0.21^b^	6.49 ± 0.35^ab^	6.24 ± 0.34^b^
	Val^*^	5.92 ± 0.45^a^	5.44 ± 0.21^a^	5.72 ± 0.32^a^	5.87 ± 0.15^a^
	Lys^*^	7.49 ± 0.26^a^	5.20 ± 0.42^c^	5.56 ± 0.26^c^	6.72 ± 0.27^b^
	Tyr	11.66 ± 0.21^a^	7.70 ± 0.13^b^	11.95 ± 0.74^a^	11.76 ± 0.36^a^
	Ile^*^	4.77 ± 0.91^a^	3.73 ± 0.22^a^	4.02 ± 0.35^a^	4.25 ± 0.17^a^
	Leu^*^	5.31 ± 0.14^a^	3.29 ± 0.07^d^	3.76 ± 0.24^c^	4.28 ± 0.32^b^
	Phe^*^	10.74 ± 0.35^a^	8.55 ± 0.46^b^	8.52 ± 0.79^b^	9.01 ± 0.16^b^
	His	5.73 ± 0.25^a^	4.61 ± 0.11^b^	4.74 ± 0.19^b^	5.40 ± 0.32^a^
	Arg	1.87 ± 0.15^a^	1.23 ± 0.18^b^	1.26 ± 0.18^b^	1.42 ± 0.20^b^
TBAA		68.12 ± 2.39^a^	51.73 ± 1.71^c^	59.84 ± 2.91^b^	62.82 ± 1.95^ab^
EAA		44.22 ± 2.05^a^	33.99 ± 1.44^c^	35.64 ± 1.93^bc^	38.19 ± 1.22^b^
TFAA		99.88 ± 3.36^a^	78.58 ± 2.74^c^	87.52 ± 4.37^b^	94.14 ± 3.85^ab^

a−d *Means within the same line with different uppercase letters differ significantly (P < 0.05)*.

* *Represents essential amino acid*.

*TUAA, total umami amino acid; TSAA, total sweet amino acid; TBAA, total bitter amino acid; EAA, essential amino acid; TFAA, total free amino acid; Asp, aspartic acid; Glu, glutamic acid; Ser, Serine; Ala, Alanine; Gly, glycine; Thr, threonine; Cys, cysteine; Met, methionine; Val, valine; Lys, lysine; Tyr, tyrosine; Ile, isoleucine; Leu, leucine; Phe, phenylalanine; His, hlstidine; Arg, argnine*.

The total amount of FAAs in the control group was 99.88 mg/kg, and the amount in the treatment group decreased by 21.33% (−18°C/−18°C), 12.37% (−40°C/−18°C) and 5.75% (−80°C/−18°C) respectively, indicating that the sample frozen at −80°C had the highest FAA retention degree, and there was no significant difference between −80°C/−18°C and the control group (*P* > 0.05), which was directly related to the degree of water loss after thawing. After thawing, the contents of Asp, Glu, Gly, Thr, Cys, Met, Lys, Tyr, Ile, Leu, Phe, His, and Arg decreased, but the retention of these FAAs in the −80°C/−18°C group was higher, which was due to the smaller ice crystals formed by its higher freezing rate, less mechanical damage to the tissue, less water loss and less loss of free amino acids.

#### 5′-Nucleotide analysis of hand grab mutton

Nucleotide is composed of purine base or pyrimidine base, ribose or deoxyribose and phosphoric acid. It has important physiological functions. At the same time, it is also an important taste substance of meat products, which can give meat products good umami characteristics. Taste nucleotides and L-glutamic acid have good synergy, which can significantly improve the umami characteristics of meat products and is also an important way to improve the umami of meat products in the process of meat processing (36, 44). 5'-GMP and 5'-IMP are important umami nucleotides. When used, the two substances are generally mixed in the ratio of 1:1. When their addition amount reaches more than 5% of Glu content, the umami characteristics of food can be significantly improved. In the processing of meat products, ATP forms ADP under the action of ATPase. Under the catalysis of phosphokinase, ADP is degraded to form AMP, further dehydrogenated to form IMP, and part of IMP forms I under the action of phosphokinase, and then further hydrolyzed to form Hx (41). It can be seen from Table 5 that the contents of 5′-AMP, 5′-GMP and 5′-IMP in the control group were significantly higher than those in the freezing group (*P* < 0.05), which might be taken away some taste substances due to the water loss of HGM after thawing. The contents of 5'-AMP and 5'-IMP in −40°C/−18°C group and −80°C/−18°C group were significantly higher than those in −18°C/−18°C group (*P* < 0.05), which may be the most water loss in −18°C/−18°C group.

**Table 5:** Taste nucleotides of HGM with different freezing rates.

**Taste substances**	**Taste attributes**	**Control group**	**−18**°**C/**−**18**°**C**	**−40**°**C/**−**18**°**C**	**−80**°**C/**−**18**°**C**
5^′^-AMP	Umami	13.22 ± 0.34^a^	10.77 ± 0.34^c^	11.99 ± 0.66^b^	12.19 ± 0.44^b^
5^′^-GMP		16.01 ± 0.77^a^	14.83 ± 0.23^b^	15.06 ± 0.27^ab^	15.18 ± 0.49^ab^
5^′^-IMP		39.54 ± 0.37^a^	24.80 ± 0.39^d^	36.56 ± 0.09^c^	37.78 ± 0.07^b^
Total		68.77 ± 1.21^a^	50.40 ± 0.78^c^	63.61 ± 0.83^b^	65.15 ± 0.82^b^

a−d *Means within the same line with different uppercase letters differ significantly (P < 0.05)*.

#### Taste activity value and equivalent umami concentration analysis of hand grab mutton

Compounds with TAV > 1 are considered to have taste activity and contribute to the overall taste, and the greater the value is, the greater the contribution value is. TAV < 1 means there is no taste activity and no contribution to the overall taste (37, 44).

TAV is the most classical and objective method to study the taste intensity of taste substances in food and the contribution of a single component to food taste (71). It can be seen from Table 6 that the TAVs calculated by all taste substances in HGM with different freezing rates were far <1. Therefore, the direct contribution of these substances to the taste of HGM was not significant. However, these taste substances can contribute to the overall flavor of HGM through synergistic effect. For example, 5'-IMP could cooperate with other free amino acids and taste nucleotides. Misako et al. (72) found that although the TAVs of amino acids and other nucleotides were <1, they could cooperate with 5'-IMP to give food strong umami. Lioe et al. (73) found that bitter amino acids below the taste threshold could enhance the freshness and sweetness of other amino acids. For example, Arg could work synergistically with NaCl and Glu to provide a pleasant overall taste. Phe and Tyr were aromatic amino acids with bitter taste, but they had been found to be important flavor components in soy sauce except Glu. Additionally, studies have shown that ASP can also act in synergy with NaCl alone (74). Chen et al. (75) studied the non-volatile taste active substances in Chinese mitten crab meat. Because the concentrations of other amino acids in crab meat were significantly lower than the corresponding taste detection threshold, it was speculated that some amino acids interacted with each other at their sub-threshold concentration to enhance the freshness and sweetness. Due to the loss of taste substances after freezing and thawing, the TAVs of the treatment groups also showed a general downward trend, and the TAVs of taste substances in samples with high freezing rate was also high.

**Table 6:** TAVs of HGM with different freezing rates.

**Taste substances**	**Threshold**	**Control group**		**−18**°**C/-18**°**C**		**−40**°**C/-18**°**C**		**−80**°**C/-18**°**C**	
	**(mg/kg)**	**Content**	**TAV**	**Content**	**TAV**	**Content**	**TAV**	**Content**	**TAV**
Asp	1,000	2.47	<0.01	1.62	<0.01	1.70	<0.01	2.35	<0.01
Glu	300	12.86	0.04	12.16	0.04	11.82	0.04	14.13	0.05
Ser	1,500	1.08	<0.01	0.85	<0.01	0.91	<0.01	1.10	<0.01
Ala	600	2.82	<0.01	2.71	<0.01	2.93	<0.01	2.65	<0.01
Gly	1,300	9.37	<0.01	7.92	<0.01	8.75	<0.01	9.27	<0.01
Thr	2,600	3.16	<0.01	1.59	<0.01	1.57	<0.01	1.82	<0.01
Cys	N	7.80	-	5.79	-	7.82	-	7.87	-
Met	300	6.83	0.02	6.19	0.02	6.49	0.02	6.24	0.02
Val	400	5.92	0.01	5.44	0.01	5.72	0.01	5.87	0.01
Lys	500	7.49	0.01	5.20	0.01	5.56	<0.01	6.72	0.01
Tyr	910	11.66	0.01	7.70	0.01	11.95	0.01	11.76	0.01
Ile	900	4.77	0.01	3.73	<0.01	4.02	<0.01	4.25	<0.01
Leu	1,900	5.31	<0.01	3.29	<0.01	3.76	<0.01	4.28	<0.01
Phe	900	10.74	0.01	8.55	0.01	8.52	0.01	9.01	0.01
His	200	5.73	0.03	4.61	0.02	4.74	0.02	5.40	0.03
Arg	500	1.87	<0.01	1.23	<0.01	1.26	<0.01	1.42	<0.01
5^′^-AMP	500	13.22	0.03	10.77	0.02	11.99	0.02	12.19	0.02
5^′^-GMP	125	16.01	0.13	14.83	0.12	15.06	0.12	15.18	0.12
5^′^-IMP	140	39.54	0.28	24.80	0.18	36.56	0.26	37.78	0.27

As shown in Table 7, the EUC of the four groups of samples was between 3.19 and 4.80 g MSG/100 g, which was much higher than the freshness threshold of MSG (30 mg/100 g). The EUC of the control group was higher than that of each treatment group. In the treatment group, the EUC of −80°C/−18°C group was the highest and the EUC value of −18°C/−18°C group was the lowest, which was jointly determined by the content of umami substances left in the mutton after thawing. Because taste nucleotides could play a synergistic role in enhancing freshness at a very low content, after thawing, the content of fresh substances in HGM was significantly reduced, and the EUC of the product was significantly reduced, which was also the reason why the EUC of the control group was significantly higher than that of the freezing group.

**Table 7:** EUC of HGM with different freezing rates.

**Sample**	**Control group**	**−18**°**C/-18**°**C**	**−40**°**C/-18**°**C**	**−80**°**C/-18**°**C**
EUC (g MSG/100 g)	4.80	3.19	3.94	4.22

Most researchers focused on umami taste in the research of meat products, free amino acids and nucleotides are important taste related compounds in meat products (76, 77). In this study, the calculation of EUC only covered the influence of umami amino acids and 5'-nucleotides on the umami of the samples. In recent years, a series of structurally diverse umami compounds have been discovered and identified, including free L-amino acids, purine nucleotides, peptides, organic acids, amides, and their derivatives (78). Umami peptides can supplement and enhance the overall taste of food, making it more harmonious, soft, and full-bodied (79, 80). In meat products, many small molecular umami peptides have been characterized and identified recently, and they also played a significant role in the overall umami of meat products (76, 77). In particular, the raw material of HGM was ribs. As one of the main components of rib, the bones might release small molecular peptides into muscle during cooking, thus affecting the umami of the samples (77).

### Sensory evaluation

The sensory panel analysis of the HGM is shown in Table 8. The control group had the highest sensory evaluation scores. The −80°C/−18°C sample had higher aroma scores than the other samples (*P* < 0.05). The sensory evaluation results for aroma were in accordance with the results of volatile flavor compound concentrations and the ROAVs of key volatile flavor compounds. The umami scores of the samples showed −80°C/−18°C > −40°C/−18°C > −18°C/−18°C (*P* < 0.05). The −80°C/−18°C sample was more acceptable than lower rate frozen samples (*P* < 0.05). The −18°C/−18°C sample had lower juiciness scores than the other samples (*P* < 0.05). The results of juiciness scores were in accordance with the results of thawing loss. Notably, the −80°C/−18°C sample had the highest overall acceptability (*P* < 0.05), which indicates that the sample with higher freezing rate had higher sensory acceptability.

**Table 8:** Sensory evaluation of HGM with different freezing rates.

**Sample**	**Aroma**	**Umami**	**Juiciness**	**Overall**
				**acceptability**
Control group	6.37 ± 0.52^a^	5.22 ± 0.25^a^	5.19 ± 0.41^a^	5.95 ± 0.63^a^
−18°C/−18°C	2.42 ± 0.85^c^	1.97 ± 0.49^d^	3.89 ± 0.98^b^	2.63 ± 0.59^b^
−40°C/−18°C	3.06 ± 0.54^c^	3.60 ± 0.12^c^	4.87 ± 0.75^a^	3.41 ± 0.20^b^
−80°C/−18°C	4.90 ± 0.81^b^	4.33 ± 0.40^b^	4.74 ± 0.26^a^	4.80 ± 0.92^a^

a−d *Means within the same raw with different uppercase letters differ significantly (P < 0.05)*.

## Conclusion

In this study, the contents of volatile compounds in high-speed frozen HGM (−80°C/−18°C group) were significantly higher than that in low-speed frozen HGM (−40°C/−18°C and −18°C/−18°C group), especially aldehydes, alcohols and ketones. For the −80°C/−18°C group, most key odor compounds had the highest ROAVs, indicating that the high freezing rate retained the typical aroma of HGM to the greatest extent. HGM samples were clearly divided into four groups, which indicated that there were significant differences in volatile composition of four groups of HGM samples. The contents of TFAAs, TSAAs, TBAAs and 5'-nucleotides in −80°C/−18°C group were significantly higher than those in −40°C/−18°C group and −18°C/−18°C group. At the same time, −80°C/−18°C group had the highest freshness due to the highest EUC. It can be concluded that the HGM frozen at a high rate (−80°C/−18°C) had the best flavor fidelity effect after short-term freezing, which was closest to fresh HGM and conducive to the fidelity of HGM flavor.

## Data availability statement

The original contributions presented in the study are included in the article/supplementary material, further inquiries can be directed to the corresponding author.

## Author contributions

Y-ZB: conceptualization, formal analysis, investigation, and writing–original draft preparation. Y-LL: validation and supervision. R-ML: supervision, project administration, and funding acquisition. CJ: writing–review and editing. SG: investigation and formal analysis. SB: methodology and resources. Y-RW: methodology and resources. F-JD: writing–review and editing. X-LH: visualization, software, and data curation. J-JG: investigation and formal analysis. All authors contributed to the article and approved the submitted version.

## Funding

This study was financially supported by the State Key Research and Development Plan Modern Food Processing and Food Storage and Transportation Technology and Equipment (2018YFD0400101).

## Conflict of interest

The authors declare that the research was conducted in the absence of any commercial or financial relationships that could be construed as a potential conflict of interest.

## Publisher's note

All claims expressed in this article are solely those of the authors and do not necessarily represent those of their affiliated organizations, or those of the publisher, the editors and the reviewers. Any product that may be evaluated in this article, or claim that may be made by its manufacturer, is not guaranteed or endorsed by the publisher.
